# Implementation of the policy protocol for management of surgical and non-surgical wounds in selected public health facilities in Ghana: An analytic case study

**DOI:** 10.1371/journal.pone.0234874

**Published:** 2020-06-23

**Authors:** Robert Kaba Alhassan, Quarshie-Odoo Benedicta Karley, Ennin Francis Ackah, Irene Adodoaji

**Affiliations:** 1 Institute of Health Research, University of Health and Allied Sciences, Ho, Ghana; 2 Department of Nursing, School of Nursing and Midwifery, University of Health and Allied Sciences, Ho, Ghana; Massachusetts General Hospital, UNITED STATES

## Abstract

**Background:**

It is estimated that millions of patients are affected by healthcare associated infections (HAIs) each year. In Ghana, high prevalence of HAIs in relation to non-surgical (also called contaminated wounds) and surgical wounds (also called sterile wounds) is largely attributed to poor adherence to policy protocols for wound management by frontline clinical staff especially nurses.

**Objective:**

Investigate the extent to which nursing staff adhere to the policy protocol for management of non-surgical and surgical wounds in selected public health facilities in Ghana.

**Methodology:**

This is an analytic case study among nursing staff (n = 140) in three government facilities in the Volta region of Ghana. Subjective and objective performance scores of staff on adherence proxies were compared using the Wilcoxon Signed-rank test, and univariate ordered logistic regression analysis used to predict staff likelihood of adherence to policy protocols on non-surgical and surgical wound management.

**Findings:**

Overall, staff self-rated themselves higher on subjective performance proxies relative to their objective scores (p<0.05). Staff with more years of work experience did not translate into a higher likelihood of adhering to standard protocol on wound management (Coef. = -0.49, CI = -0.93–0.05, p = 0.036). Being a senior nursing officer relative to lower nursing ranks increased staff likelihood of complying particularly with standard policy protocol for management of non-surgical wounds (Coef. 5.27, CI = 0.59 9.95, p = 0.027).

**Conclusion:**

There is the need for accelerated in-service training for staff on standard protocols for wound management coupled with supportive supervisions. Staff adherence to standard quality care protocols should be a pre-requisite for licensing of health facilities by regulatory bodies like Health Facilities Regulatory Agency and National Health Insurance Authority.

## Background

According to a World Health Organization (WHO) report [1], infections associated with the continuum of healthcare delivery are the most frequent adverse medical events proven to be detrimental to the safety of patients globally. Even though the global burden of Healthcare Associated Infections (HAIs) remains unknown due to data constraints, it is estimated that hundreds of millions of patients are affected by HAIs each year [1]. HAIs continue to contribute significantly to high mortality rates and financial losses in many health systems across the globe. Unfortunately, resource limited countries in Africa often bear the brunt of this existing challenge. The WHO further estimates that “of every 100 hospitalized patients at any given time, seven (7) in developed and fifteen (15) in developing countries will acquire at least one HAI” [1, p 21]. Even more worrying is the fact that the burden of HAIs is 2–3 times higher in under-developed countries relative to developed countries [1].

Patients who have a loss in skin integrity in the form of a surgical or non-surgical wound are particularly more prone to HAIs due to their vulnerability to infections [2]. A study conducted in ten hospitals on point prevalence of HAIs in Ghana found that out of the 2,107 inpatients surveyed, 184 HAIs were found among 172 patients, representing an overall prevalence of 8.2% with the most common HAIs being surgical site infections [3]. Other similar studies on Ghana [4–9] and elsewhere [10, 11] have equally alluded to high prevalence of HAIs related to wound infections be they non-surgical or surgical. This study operationally defined a surgical wound as a break in the skin tissue integrity created under sterile conditions such as surgical incisions and are without infections. Non-surgical wound on the other hand is skin dis-integrity created under unsterile conditions including clean-contaminated, contaminated, or dirty wounds but can be repaired surgically.

Given the increasing burden of HAIs on healthcare systems and the compelling evidence of their effect on patient safety and overall health outcomes, policy makers and health regulators have prioritized strategies for strict adherence to standards of care for patients at higher risk of acquiring HAIs. Adherence to standard protocols on care of patients with wounds has the propensity of reducing patients’ exposure to HAIs and enhance prognosis of patients’ condition.

In order to promote desired outcomes for patients with surgical and non-surgical wounds, healthcare professionals involved in their care and treatment are expected to have the requisite knowledge and skills on the standard policy protocols in wound management and accordingly apply these skills as expected of them. Unfortunately, over the years there has been a gap between policy and practice in wound management, especially among nursing and other clinical staff who often perform these important clinical roles in Ghana [12–14].

A study conducted by McCluskey and McCarthy [15] revealed that nurses’ knowledge on wound management does not always reflect in their clinical practice. This gap between policy and practice often results in avoidable instances of wound complications which are the third most common HAIs in many clinical environments [1]. A study conducted among nurses in a University Hospital in Brazil, found that 93% of them had inadequate knowledge of the standard protocol for wound management and about 30% of all surgical procedures ended up getting infected [16].

The economic cost of poor management of wounds due to wide policy practice gaps is enormous. Even though there are no many empirical figures on Ghana and Africa at large, anecdotal data suggests the situation is generally worst in resource constrained settings [1]. Data on Sub-Saharan Africa (SSA) estimates that HAIs resulting in neonatal sepsis alone accounts for 5.29–8.73 million lost DALYs with a predicted annual economic burden ranging from $10 billion to $469 billion [17].

In light of the dire medical and economic effects of non-adherence to standard protocols in the management of wounds, it is imperative existing policy practice gaps are closed. Even though the policy—practice gap in the management of wounds remains a critical public health concern to many health systems across the globe, there is paucity of empirical data, especially in resource limited settings such as Ghana. This study therefore sought to investigate the extent to which clinical nursing staff adhere to the policy protocol for non-surgical and surgical wounds in selected public health facilities in Ghana and the determinants, using the Nursing and Midwifery Council of (NMC) Ghana guidelines for wound management. In the context of this paper, the NMC, Ghana recognized terminologies are used where non-surgical wounds are also referred to as contaminated wounds and surgical wounds also referred to as sterile wounds.

## Methods

### Study design

The study was an analytic case study involving professional and auxiliary clinical nursing staff from selected government hospitals in the Ho Municipality of the Volta region of Ghana. The study was conducted between 28^th^ February and 20^th^ March, 2019.

### Study setting

The Volta region is one of the sixteen (16) administrative regions in Ghana with 2019 project population size of 1,865,332 million people, representing approximately 6% of the estimated 30,380,482 million people in Ghana [18]. Geographically, the Volta region is along the eastern coast of the Gulf of Guinea to the south, Republic of Togo to the east, Oti region to the north and the Greater Accra and Eastern regions to the west [18]. The 2019 annual population projection for the Ho Municipality is 218,948 which constitutes approximately 12% of the total regional population [18].

### Health indicators in study setting

The Volta region has a total of 731 healthcare facilities comprising of one (1) teaching hospital, 30 hospitals, 45 clinics, 156 health centres, 14 maternity homes, 4 polyclinics and 482 Community-based Health Planning and Services (CHPS) compounds. The CHPS compounds in Ghana are the basic unit of primary healthcare delivery at the community level [18]. In terms of bed capacity, the Volta region share of the national figure of 30,272 is 8.3% with government-owned facilities recording 2,217 beds with a bed utilization rate of 55.1 as at 2018 compared to the national average of 58.3 [18].

In 2018 a total of 74,065 clinical and non-clinical health professionals were working under the Ghana Health Service (GHS) at various levels across all the administrative regions in Ghana. Out of this number 6,709 worked in the Volta region, representing approximately 9% of the total workforce. Moreover, the doctor to patient ratio in the Volta region in 2018 was 1: 11, 857 compared to the national average of 1: 7,058 [18]. Out of the 21,374 registered general nurses working under the GHS barely 1,455 of them were working in the Volta region, representing a regional percentage share of 12%; nurse to population ratio in the Volta region in 2018 was estimated to be 1: 567 relative to the national average of 1: 508 [18].

In terms of service output, the Volta region recorded 2,454,247 outpatient department (OPD) attendance in 2018 out of the national figure of 30,852,581 with an OPD per capita of 0.96 relative to the national figure of 1.0. According to the WHO Health Systems Strengthening (HSS) handbook (2010), outpatient department (OPD) visits per capita are the number of visits to health care facilities per capita (per head), including repeat visits (excluding immunizations) during one year relative to the total population of the same geographical area. Health care facilities include all public, private, non-governmental and community-based health facilities in which general health services are offered. The indicator is sometimes used as a proxy measure for patients’ access to health care. Inpatient attendance on the other hand was 145,404 for the Volta region out of the national figure of 1,642,023. Hospital admission rate was 56.9 against the national average of 55.4 in the same year [18]. According to the WHO global reference list for 100 core health indicators (2015), hospital admissions rate is the number (and mean) of hospital admissions per person per year. Indicator formula is: Number of hospital admissions per year/Total population of the same geographical area. It is sometimes used as a measure of quality care outcome.

### Study population

Three public health facilities within the Ho Municipality were purposely selected for this study. These facilities were selected along the spectra of tertiary, secondary and primary health facilities as per the Ghana Health Service (GHS) pyramidal levels of healthcare and referral system. The tertiary level facility had a staff strength of 651. Out of this figure, 262 were nursing staff comprising of 69 midwives, 11 mental health nurses, 16 community health nurses and 113 general nurses. Staff strengths were relatively lower in the secondary and primary level healthcare facilities.

**Fig 1:**
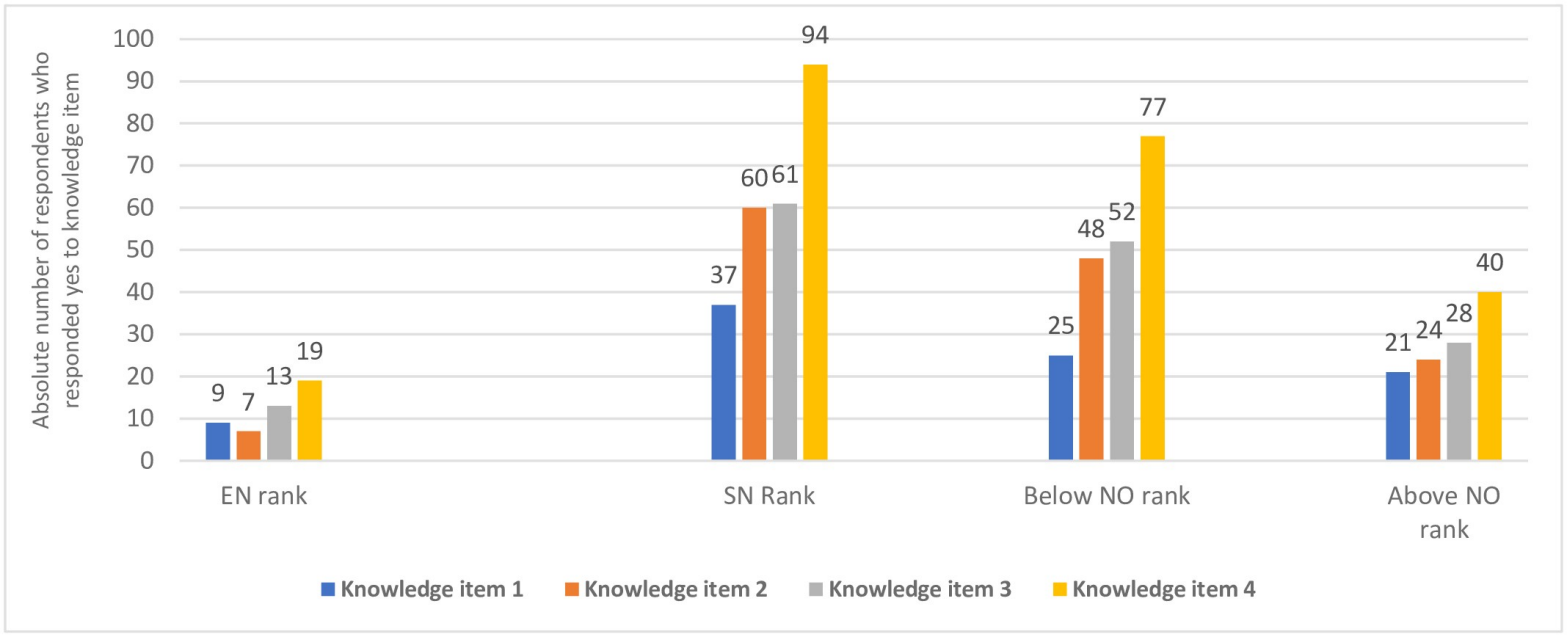
Staff knowledge on basic wound dressing standard policy protocols (n = 140). Field Data (2019); Knowledge factor 1 (Staff who knew to the use of methylated spirit and povidone iodine for surgical wound care), Knowledge factor 2 (Staff who knew the use of normal saline and povidone iodine for septic wound care), Knowledge factor 3 (Staff who knew when a trolley is bathed before wound care), Knowledge factor 4 (Staff who knew they have to wash their hands at least two-times before wound dressing); SN (Staff Nurse); NO+ (Nursing Officer or higher professional rank); NO- (Below the ranks of a Nursing Officer in professional rank); EN (Enrolled Nurses).

The study population consisted of mainly clinical nursing staff from the three selected health facilities within the Ho Municipality. The clinical nursing staff comprised of professional and auxiliary nurses in the categories of general nursing, midwifery, community health nursing and mental health nursing. Fig 1 illustrates the categories and ranks of clinical nurses in Ghana.

### Inclusion and exclusion criteria

#### Eligible participants

The study included only clinical nursing staff from the selected government hospitals in the Ho Municipality. The clinical nursing staff included registered general nurses; registered community nurses; registered mental nurses and registered midwives. These cadres were included because their routine clinical responsibilities included management of surgical and non-surgical wounds.

#### Ineligible participants

Other cadre of clinical staff who are not within the nursing fraternity were excluded because they were not within the scope of this study; moreover, wound management is predominantly performed by nurses and sometimes by auxiliaries under supervision. Additionally, student nurses, part time nurses, nurses on clinical attachment or internship were excluded because they were not likely to have sufficient institutional memory of the hospital policy protocols in wound management. Nurse trainees are not allowed to perform advanced nursing procedures like wound dressing without supervision because they do not yet have the license to practise. Nonetheless, there are isolated instances when nurse trainees are left alone to perform advanced nursing procedures due to shortage of nurses demonstrated earlier in respect of the appalling nurse: patient ratios.

### Sampling and sample size determination

Multi-staged sampling technique was employed involving quota and purposive sampling. The sample size for the study was determined using the Krejcie and Morgan [19] formula for determining sample size based on known populations. Since the population of target clinical nursing staff was known, the representative sample sizes in the three health facilities were determined as follows: tertiary facilities (n = 120), secondary facilities (n = 60), and primary facilities (n = 20). A non-response rate of 5% was added to the target sample of 200 resulting in a total sample size of 210. Krejcie and Morgan formula is stated as follows: S = (X^2 NP(1-P))/(d^2 (N-1) + X^2 P(1-P)), where:

- S is required sample size
- *X^2 is the table value of chi-square for 1 degree of freedom at a desired confidence level (3*.*841)*
- N is the population size
- *P is the population proportion (assumed to be 0*.*50 since this would provide the maximum sample size)*
- *d is the degree of accuracy expressed as a proportion (0*.*05)*

### Instrument of data collection and components

#### Subjective assessment component

The data collection instrument had two main components for assessing the respondents’ perceived/subjective and objective adherence to the policy protocol on wound management. The subjective assessment comprised of a structured questionnaire of open and close ended questions. The questionnaire was sub-divided into Section A (Socio-demographic and work history of participants which includes the age, sex, religion, occupation, professional category, rank and work experience); Section B (participants’ knowledge of the standard policy protocol for wound management); Section C (subjective and objective assessments on policy protocol for surgical wound management on an adapted five-point Likert scale 0 = “very rarely adherent” to 4 = “all the times adherent”); Section D (subjective and objective assessments on policy protocol for non-surgical wound management also on an five-point Likert scale); Section E (staff constraints in adhering to standard policy protocol for wound management and recommendations).

#### Objective assessment component

Besides the subjective assessment, an objective assessment was done to determine the level of adherence of respondents to the standard policy protocol on wound management using the same assessment criteria in sections C and D. The objective assessment was done for one respondent at a time by two independent researchers using the same adapted five-point Likert scale from 0 = “very rarely adherent” to 4 = “all the times adherent”. Average score of the two assessors was then agreed on and computed for the assessed participant. Total number of questions (assessment criteria) on non-surgical wound management were 14 while the questions on surgical wound management were 16. The objective assessments were meant to serve as control for the subjective assessments scores and did not necessarily constitute the crux of the study since the number of respondents assessed objectively was relatively small to inform meaningful statistical analysis.

The data collection tool was developed by the researchers and not under copyright more restrictive than CC-BY hence a copy of the data collection tool (see S1 File) has been attached as supplementary material to promote replication of analysis by future researchers.

### Validity and reliability

The Nursing and Midwifery Council (NMC) policy protocols for wound management was used in this study and these tools are already validated and being applied in Ghana. Additionally, the staff questionnaires were validated through piloting and peer reviews guided by empirical literature on nursing protocols for surgical and non-surgical wound management. Where appropriate, components of validated instruments were adapted to suit the clinical nursing context in Ghana. Pre-testing was done in one hospital within the Ho Municipality to promote clarity and avoid ambiguity of questions.

### Data collection procedure

Designed questionnaires were employed to collect primary data from eligible study participants who willingly consented to participate in the study. Independent objective assessment of participating nursing staff was conducted using adapted NMC of Ghana standard policy protocol on wound management in addition to the self-rated adherence. The subjective questionnaires were self-administered by the eligible respondents and later retrieved by trained research assistants. The objective assessments on the other hand were administered by two research assistants concurrently per respondents to avoid potential bias by the researchers. Assessments were done on the ward after voluntary consent by participants who knew they were being assessed for adherence to the policy protocols on wound management and later debriefed on their scores.

### Ethics approval and consent to participate

Research Ethics Committee (REC) of the University of Health and Allied Sciences (UHAS) specifically approved this study with clearance number: UHAS-REC A.4[332]18–19. Individual informed consents were also sought from the respondents as well as administrative approvals from the sampled health facilities. Anonymity of respondents was assured through coding.

### Data analysis

Field data was analyzed using the STATA software (version 12.0). A total of 210 structured questionnaires were administered to eligible respondents out of which 140 were completed and retrieved, representing a return rate of 67%. The data was subsequently disaggregated into two sub-samples, namely respondents who were subjectively assessed and later followed for objective assessments (n = 30, 21%), and participants who were mainly assessed subjectively (n = 110, 79%) and efforts to follow them for the objective assessments proved futile due to challenges in work schedules.

Field data was cleaned and coded for anonymity using Microsoft Excel and then exported to STATA (version 12.0) statistical analysis software for descriptive and inferential analysis. Descriptive analysis was mainly for the background information of respondents for numerical and categorical variables related to age, gender, years of work experience, professional category, rank and religious affiliation.

Performance scores of respondents were decomposed into objective and subjective mean scores and compared using the Wilcoxon Signed-rank test since the assessment scales were all on five-point Likert Scale from 0 = “very rarely adherent” to 4 = “all the times adherent”. Test of the null hypothesis was determined at 95% confidence level. The tests were run separately for non-surgical and surgical wound management procedures. In the subjective and objective assessment tools, the number of questions (assessment criteria) on management of non-surgical wounds were 14 while the number of questions on management of surgical wounds were 16. Scale reliability of the objective assessment items for surgical wound dressing was checked using Cronbach’s alpha and the scale reliability coefficient was found to be 0.78 compared to 0.80 for non-surgical wound dressing items. The scale reliability coefficient for the subjective assessment on surgical wound management was 0.43 relative to 0.38 in the case of the non-surgical wound management criteria scores.

Univariate ordered logistic regression analysis was conducted using the 140 subjective assessment data set. The objective assessment data set was not used because the data contained 30 observations which were deemed statistically inadequate to run regression analysis [20]. Univariate ordered logistic regression analysis was conducted in two different model specifications where the main outcome variables were “Overall score on non-surgical wound management” and “Overall score on surgical wound management”. These variables were defined as the cumulative score of respondents on non-surgical and surgical wound management respectively.

Explanatory (independent) variables fitted in the two models were: respondents’ age (numerical), years of work experiences (numerical) and gender (males = 1, 0 = otherwise). Other co-variates fitted in the regression model were professional categories of respondents as follows: (1 = principal nursing officer, 0 = otherwise), (1 = senior nursing officer, 0 = otherwise), (1 = nursing officer, 0 = otherwise), (1 = senior staff nurse, 0 = otherwise), (1 = staff nurse, 0 = otherwise), (1 = senior enrolled nurse, 0 = otherwise), (1 = enrolled nurse, 0 = otherwise), (1 = principal enrolled nurse, 0 = otherwise). In the first model, the variable “senior enrolled nurse” was dropped for multicollinearity while “principal nursing officer” was used as the control or reference point for the cluster of professional ranks. In the second model “nursing officer” was dropped for multicollinearity while “principal nursing officer” was maintained as the reference. Statistical significance was determined in both models at 95% confidence level while a p-value ≤ 0.05 was deemed statistically significant.

## Findings

### Background information of respondents

Out of the 210 questionnaires administered per the study sample size, 140 completed questionnaires were retrieved, representing approximately 67% return rate. Out of the 140 staff contacted for subjective assessment of their adherence to standard wound management policy protocol, only 30 (21%) were successfully followed for their corresponding objective assessment. Differences in duty schedules of staff and mobility of staff between wards created practical challenges in following up all 140 participants who initially responded to the subjective assessment questions coupled with limited time and financial resources to conduct a longitudinal study design which would have been more beneficial in getting higher responses from the objective assessments.

The field data showed that 70% of the respondents were females and nearly 100% of them said their religious affiliation is Christianity. In terms of professional rank of the respondents, the dominant rank was “Senior Staff Nurse” (22%) followed by Staff Nurses (21%) and Nursing Officers (20%). The least professional rank was “Principal Nursing Officer” (3%). Registered general nurses constituted the largest percentage of staff interviewed (84%), followed by registered midwives (7%), registered community nurses (6%) and registered mental nurses (3%). Average age of respondents was 30 (SD = 6.1) while the average years of work experience was 5.2 (SD = 5.1) (see Table 1).

**Table 1:** Socio-demographics and work history of respondents.

Characteristics	Statistic		
	*Objective (n = 30)	**Subjective (n = 110)	Total (n = 140)
**Gender (n = 140)**	f (%)	f (%)	f (%)
Male	9(6)	33(24)	42(30)
Female	21(15)	77(55)	98(70)
**Religion (n = 140)**			
Christians	30(22)	108(77)	138(99)
Other religions	0(0)	2(1)	2(1)
**Professional rank (n = 128)**			
Principal nursing officer	2(1.5)	2(1.5)	4(3)
Senior nursing officer	1(1)	12(9)	13(10)
Nursing officer	5(4)	21(16)	26(20)
Senior staff nurse	8(6)	21(16)	29(22)
Staff nurse	7(5)	20(16)	27(21)
Senior enrolled nurse	3(2)	14(11)	17(13)
Enrolled nurse	2(1)	10(8)	12(9)
**Professional category (n = 123)**			
General nurses+	22(18)	81(66)	103(84)
Midwives	4(3)	5(4)	9(7)
Mental nurses	1(1)	3(2)	4(3)
Community nurses++	0(0)	7(6)	7(6)
	**Mean ±SD**	**Mean ±SD**	**Mean ±SD**
Age (n = 122)	31.3±7.8	29.9±5.5	30.2±6.1
Years of work experience (n = 132)	6.4±7.0	4.9±4.4	5.2±5.1

Field Data (2019);

*Objective assessments (staff who were successfully assessed both objectively and subjectively on the standard policy protocol on wound management);

**Subjective assessments (staff who were only subjectively on the standard policy protocol on wound management and were lost to objective assessment); SD (Standard Deviation);

^+^Includes 4 senior enrolled nurses and 11 senior enrolled nurses who are auxiliaries;

^++^includes 1 enrolled nurse who is an auxiliary nurse.

### Staff knowledge on basic wound management policy protocols

**Fig 2:**
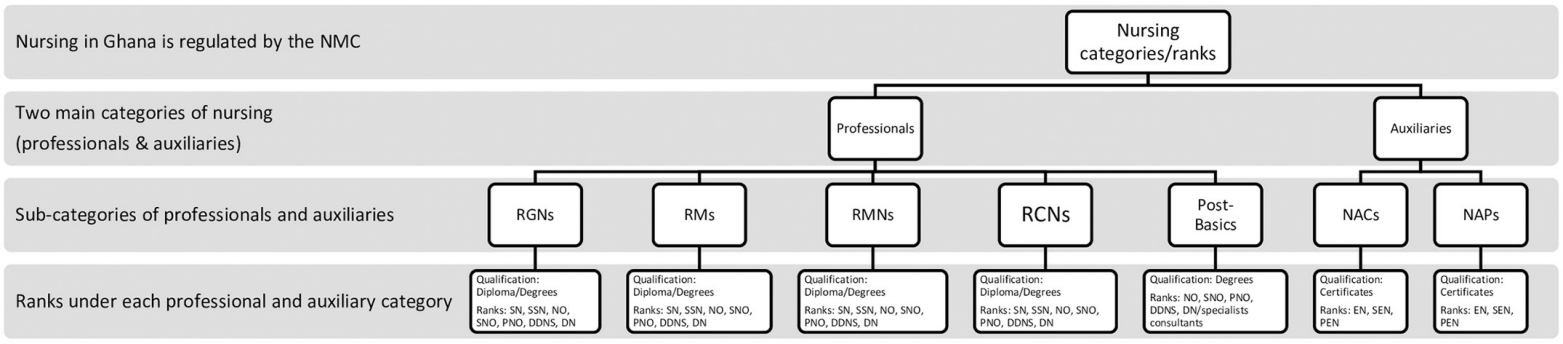
Categories and ranks of clinical nurses in Ghana. NMC (Nursing and Midwifery Council); RGNs (registered general nurses); RMs (registered midwives); RMNs (registered mental nurses); RCNs (registered community nurses); NACs (nurse-assistant clinicals); NAPs (Nurse-assistant preventive); EN (enrolled nurse); SEN (Senior enrolled nurse); PEN (principal enrolled nurse); Post-basics (these are specialized post-basic diploma or first degree courses and in some cases post-graduate specialist courses at the Ghana college of Nurses and Midwives; Courses under the category of post-basics include: public health nursing, ear-nose and throat nursing, critical and emergency case nursing, perioperative and theatre nursing, ophthalmic nursing, midwifery).

Four routine standard practices on wound management were used as proxies to determining staff knowledge of the policy protocol on wound management in accordance with the Nursing and Midwifery Council (NMC) of Ghana standard guidelines. The knowledge items were scored for each staff in a binary outcome, thus either “a staff had knowledge” or otherwise (i.e. Yes or No). The results showed that majority of staff had knowledge of the protocol on hand washing at least two-times before wound dressing followed by the protocol on when a trolley is bathed before wound dressing. The least known protocol guidelines were “use of methylated spirit and povidone iodine for surgical wound management”, and “use of normal saline and povidone iodine for non-surgical wound management”. There was no significant difference between the professional categories with respect to knowledge of these standard protocols (see Fig 2).

### Staff performance on care of non-surgical wounds: Comparing objective and subjective scores

Apart from the knowledge items on wound management, the researchers also explored staff compliance with specific standard guidelines on care of non-surgical and surgical wounds. Total of fourteen (14) standard guidelines were used to measure adherence at the level of staff objectively and subjectively. With respect to the non-surgical wound management it was found that staff self-rated their performance highest in the areas of “preparation of trolley to patient bedside” ([subjective scores: mean = 4.0, CI = 3.9 4.0] objective scores: mean = 3.1, CI = 2.6 3.6]) and “cleaning wound with series of swaps until wound is clean” ([Subjective scores: Mean = 4.0, CI = 3.9 40.0], Objective scores: mean = 3.4, CI = 3.1 3.7]).

Other self-rated area with high scores was “application of sterile dressings using prescribed lotion” ([Subjective scores: mean = 3.9, CI = 3.8 4.0], Objective scores: mean = 3.5, CI = 3.3 3.8]). The components with the least self-ratings were “irrigation of wound with saline from within outwards” ([Subjective scores: mean = 3.3, CI = 3.0 3.6], Objective scores: mean = 1.8, CI = 1.1 2.6]) and “making patient comfortable in bed and explaining findings of wound dressing to him/her” (Subjective scores: mean = 3.5, CI = 3.2 3.8], Objective scores: mean = 1.0, CI = 0.5 1.5]). Overall, the self-rated scores by staff were higher than their corresponding objective assessment scores by independent assessors (p<0.05) (see Table 2).

**Table 2:** Comparison of subjective and objective scores of staff for standard protocol on non-surgical wounds (n = 26).

Standard protocol	Performance scores		
	Subjective Score	Objective Score	p-value*
	Mean [95% CI]	Mean [95% CI]	
1. Explains procedure to patient and ensure privacy	3.8 [3.6 4.1]	1.4 [0.8 1.9]	0.0000
2. Prepares and takes trolley to the patient’s bedside	4.0 [3.9 4.0]	3.1 [2.6 3.6]	0.0002
3. Positions patient comfortably and protects bedclothes	3.8 [3.7 4.0]	1.4 [2.1 3.1]	0.0001
4. Exposes area of wound and removes plaster or bandage	4.0 [3.9 4.0]	3.3 [3.1 3.5]	0.0000
5. Washes and dry hands, assembles instruments and pour lotion into gallipot	3.8 [3.7 4.0]	2.9 [2.5 3.3]	0.0002
6. Removes soiled dressing with dissecting forceps or gloved hand, discards, washes	3.6 [3.3 3.9]	2.9 [2.5 3.3]	0.0142
7. Dabs or cleans wounds with sterile or gloves using prescribed lotion or gently	3.8 [3.5 4.0]	3.2 [2.8 3.5]	0.0015
8. Irrigates cleaned wound with syringe and saline from within outward and cleans the surrounding skin	3.3 [3.0 3.6]	1.8 [1.1 2.6]	0.0057
9. Cleans wound with series of swaps until its clean	4.0 [3.9 4.0]	3.4 [3.1 3.7]	0.0006
10. Applies sterile dressing using prescribed dressing lotion and secures into position or leaves exposed where necessary	3.9 [3.8 4.0]	3.5 [3.3 3.8]	0.0185
11. Makes patient comfortable in bed, explains relevant findings to patient and thank him	3.5 [3.2 3.8]	1.0 [0.5 1.5]	0.0000
12. Discards trolley and decontaminates used instruments and wash hands	3.8 [3.6 4.1]	3.3 [3.1 3.5]	0.0007
13. Removes gloves and screens, washes and dries hands	3.8 [3.6 4.1]	3.0 [2.6 3.4]	0.0017
14. Documents and reports state of the wound	3.6 [3.3 3.9]	1.1 [0.5 1.7]	0.0000
15. Overall score	3.8 [3.7 3.9]	2.6 [2.4 2.8]	0.0000

Source (Field Data, 2019);

*Wilcoxon signed-rank test of null hypothesis statistically significant (p<0.005); SACS (Subjective Surgical Clinical Scores); OSCS (Objective non-surgical Clinical Scores)

Standard guidelines that objectively scored worst were: “making patient comfortable in bed and explaining findings of wound dressing” (mean = 1.0, CI = 0.5 1.5) followed by “documentation on state of wound after procedure” (mean = 1.1, CI = 0.5 1.7). Other worst rated areas, objectively, are “explaining procedure to patient and ensuring privacy” (mean = 1.4, CI = 0.8 1.9) and “positioning patient and covering bedclothes during procedure” (mean = 1.4, CI = 2.1 3.1). The overall subjective/self-rated score by staff was 3.8 (CI = 3.3 3.9) compared to the overall objective score of 2.6 (CI = 2.4 2.8) (p = 0.0001).

### Staff performance on care of surgical wounds (comparing objective and subjective scores)

In terms of staff performance on adherence to standard protocol for surgical wound dressing, it was found that staff self-rated themselves highest compared to their corresponding objective scores in the areas of “establishment of rapport and explaining procedure to patient” ([Subjective scores: mean = 3.9, CI = 3.7 4.0], Objective scores: mean = 1.7, CI = 1.2 2.3, p = 0.0001]), “cleaning wound with series of swaps until wound is clean” ([Subjective scores: mean = 3.9, CI = 3.7 4.0], Objective scores: mean = 3.9, CI = 3.2 3.4], p = 0.0002]) and “application of sufficient sterile dressings and securing into position” ([Subjective scores: mean = 3.9, CI = 3.8 4.0], Objective scores: mean = 3.7, CI = 3.5 3.8], p = 0.0196]).

Similar to results on the non-surgical wound guidelines, it was found that the worst objective scores were on components such as “documentation and reporting on state of wound” (mean = 0.9, CI = 0.4 1.4), “informing patient on state of wound” (mean = 0.8, CI = 0.3 1.3), and “thanking patient and making him/her comfortable in bed after procedure” (mean = 1.5, CI = 1.0 2.0). The overall staff self-rated/subjective score was 3.7 (CI = 3.5 3.8) compared to the overall objective score of 2.6 (CI = 2.5 2.7), p = 0.0001) (see Table 3).

**Table 3:** Comparison of subjective and objective scores of staff for standard protocol on surgical wounds (n = 30).

Standard protocol	Performance scores		
	Subjective Score	Objective Score	p-value*
	Mean [95% CI]	Mean [95% CI]	
1. Establishes rapport and explains procedure to patient	3.9 [3.7 4.0]	1.7 [1.2 2.3]	0.0000
2. Put on mask, prepares and takes trolley to bedside and provides privacy	3.7 [3.5 4.0]	2.5 [1.9 3.0]	0.0001
3. Ask assistant to put patient into desired position, protect bed cloth and expose area.	3.6 [3.4 3.8]	2.7 [2.4 3.0]	0.0001
4. Ask assistant to pour out lotions into gallipot	3.8 [3.6 4.0]	3.1 [3.0 3.2]	0.0000
5. Ask assistant to remove plaster or bandage	3.4 [3.0 3.7]	3.2 [3.0 3.4]	0.1131
6. Remove soiled dressing with dissecting forceps or disposable gloves and discard	3.2 [2.6 3.7]	3.2 [3.0 3.4]	0.4585
7. Wash and dry hands and wear sterile gloves or use sterile forceps	3.7 [3.4 4.0]	2.3 [1.8 2.8]	0.0001
8. Clean wound with swaps soaked in normal saline using sterile forceps or sterile gloves	3.7 [3.4 4.0]	3.2 [3.0 3.4]	0.0001
9. starting from the wound outward using one swap at a time.	3.5 [3.3 3.8]	2.9 [2.6 3.3]	0.0109
10. Cleans wound with series of swaps until clean	3.9 [3.7 4.0]	3.2 [3.0 3.4]	0.0002
11. Apply sufficient sterile dressing and secure into position	3.9 [3.8 4.0]	3.7 [3.5 3.8]	0.0196
12. Inform patient about state of wound	3.2 [2.8 3.6]	0.8 [0.3 1.3]	0.0001
13. Thank and make patient comfortable in bed	3.7 [3.5 3.9]	1.5 [1.0 2.0]	0.0000
14. Discard trolley, decontaminate used items and remove gloves	3.8 [3.6 4.0]	3.3 [3.1 3.4]	0.0006
15. Wash and dry hands and remove screen	3.9 [3.7 4.0]	3.4 [3.2 3.6]	0.0010
16. Document and report state of wound	3.6 [3.2 4.0]	0.9 [0.4 1.4]	0.0000
17. Overall score	3.7 [3.5 3.8]	2.6 [2.5 2.7]	0.0000

Source (Field Data, 2019);

*Wilcoxon signed-rank test of null hypothesis statistically significant (p<0.005); SACS (Subjective Surgical Clinical Scores); OSCS (Objective non-surgical Clinical Scores)

### Predictors of staff adherence to standard policy protocols for wound management

Overall, summative scores of staff performance on non-surgical and surgical wound management were derived from the 140 subjective assessment data set. The two overall summative scores were modelled as the main outcome variables of interest measured in a five-point Likert scale from 0–4 where higher scores depict better staff adherence and *vice versa*. The explanatory variables in the Univariate ordered logistic regression model were respondent’s age, gender, years of work experience, and professional rank. Explanatory variables with variance inflation factors (VIFs) above 10.0 were dropped from the model for multicollinearity (see Table 4).

**Table 4:** Model specification for univariate ordered logistic regression test on adherence to standards in non-surgical and surgical wound management.

Variables	Obs.	Variable Definition	RGNs (n = 103)	Other nurses (n = 21)	p-value
			Mean (SD)	Mean (SD)	
**Outcome variables**					
Overall score on septic wound management	24	Cumulative score for septic wound management	3.22(0.26)	3.16(0.22)	0.5738*
Overall score on surgical wound management	28	Cumulative score for surgical wound management	3.15(0.20)	3.09(0.20)	0.4202*
**Independent variables**	**Obs**.	**(Co-variates)**			
Age	107	Age of staff in years	30.40(5.56)	30.25(8.79)	0.9221*
Work experience in years	118	Staff work experience in years	5.22(4.29)	6.26(8.94)	0.4332*
Sex	124	1 if male 0 if female	35(28%) 68 (55%)	7(6%) 14(11%)	0.954**
**Professional rank**					
Principal nursing officer	4	1 if principal nursing officer, 0 otherwise	3(3%)	1(1%)	0.742**
Senior nursing officer	13	1 if senior nursing officer, 0 otherwise	11(10%)	2(2%)	
Nursing officer	26	1 if nursing officer, 0 otherwise	20(18%)	6(5%)	
Senior staff nurse	29	1 if senior staff nurse, 0 otherwise	25(22%)	4(4%)	
Staff nurse	26	1 if staff nurse, 0 otherwise	22(19%)	4(4%)	
Senior enrolled nurse	11	1 if senior enrolled nurse, 0 otherwise	11(10%)	0(0%)	
Enrolled nurse	5	1 if enrolled nurse, 0 otherwise	4(4%)	1(1%)	

Source (Field Data, 2019); RGNs (Registered General Nurses); SD (Standard Deviation);

*Independent Student t-test not significant at 95% confidence level;

**Pearson Chi-square test not significant at 95% confidence level.;

Obs. (number of valid observations); note: all percentages have been rounded-up to the nearest decimal.

The analysis revealed that years of work experience is a significant negative predictor of staff overall adherence to standard protocol for non-surgical wound dressing. Thus, increasing years of work experience reduces the log likelihood of staff adhering to standard protocol on non-surgical wound healing (Coef. = -0.49, CI = -0.93–0.05, p = 0.036). Being a senior nursing officer relative to other professional ranks increases a staff’s log likelihood of complying with standard policy protocol for non-surgical wound management (Coef. 5.27, CI = 0.59 9.95, p = 0.027). On the other hand, auxiliary nurses had a negative log likelihood of adhering the standard protocol for non-surgical wound management (Coef. = -4.32, CI = -8.39–0.24, p = 0.038), and surgical wound management (Coef. = -4.82, CI = -9.32–0.29, p = 0.037), relative to other professional ranks (see Table 5).

**Table 5:** Determinants of staff adherence to standard protocols on non-surgical wound management.

	Dependent variables							
Independent variables	Univariate model 1				Univariate Model 2			
	Overall non-surgical wound management				Overall surgical wound management			
	Coef.	[95%Conf.	Intv.]	p-value	Coef.	[95%Conf.	Intv.]	p-value
Age	0.24	-0.10	0.59	0.163	0.04	-0.28	0.36	0.819
Years of work experience	-0.49	-0.93	-0.05	0.030^§^	-0.10	-0.49	0.28	0.599
**Sex**								
Male	-0.54	-2.84	1.77	0.649	-0.12	-1.86	1.62	0.889
Female	1.0	1.0		1.0	1.0	1.0		1.0
**Professional rank**								
Senior nursing officer	5.27	0.59	9.95	0.027^§^	-0.19	-4.11	3.72	0.923
Nursing officer**	-.048	-3.35	2.40	0.745				
Senior staff nurse	-1.21	-3.81	1.40	0.364	-0.94	-3.48	1.59	0.466
Staff nurse	-0.71	-3.91	2.49	0.663	-1.10	-4.29	2.08	0.498
Senior enrolled nurse*					-1.26	-4.46	1.93	0.438
Enrolled nurse	-4.32	-8.39	-0.24	0.038^§^	-4.81	-9.32	-0.29	0.037§
Principal nursing officer	1.0	1.0		1.0	1.0	1.0		1.0
**Model fit statistics**								
Obs.	21				25			
LR chi2(8)	9.94				6.68			
Prob > chi2	0.2695				0.5715			
Pseudo R2	0.0910				0.0549			
Log likelihood	-49.602515				-57.470947			

Source (Field Data, 2019);

*Dropped from model 1 for multicollinearity;

**Dropped from model 2 for multicollinearity;

^§^Ordered logistic regression statistically significant (p<0.05)

## Discussion

Strict adherence to standard guidelines in wound management helps maintain the rule of asepsis and decreases the risk of contamination of the wound or transmission of organism from one patient to another [21]. Unfortunately, many healthcare facilities in resource constrained countries particularly in Africa continue to battle with this important patient safety standard. In Ghana, increasing number of days of hospital admissions have been partly blamed on HAIs which wound infections often constitute a greater share of this public health concern [22, 23].

In light this, the regulatory bodies for nurses and midwives in Ghana, the NMC and Ministry of Health (MoH), have over the years instituted, by law, professional policy guidelines for all clinical nursing procedures including wound management [24]. Sadly, adherence to these policy protocols to the latter remains a mirage in many healthcare facilities, especially those owned by government. Beyond the financial and infrastructural limitations confronting many of these health facilities, several empirical studies have pointed to wanton nonadherence to these professional guidelines on the part of clinicians, particularly nursing staff who turn to perform more of these clinical roles [12, 25].

Ghana’s efforts towards attaining the United Nations Sustainable Development Goals (SDGs) 3&4 will remain a dream if existing gaps in quality of healthcare, patient safety and risk reduction in clinical settings are not closed. Since, nursing staff constitute over 50% of the workforce in Ghana’s healthcare system [18], their professional actions and inactions turn to have deleterious effect on many health outcome indicators including incidence and prevalence of HAIs [12]. In view of this, the investigators sought to examine the current practices of nurses in the management of wounds and determine factors associated with adherence or otherwise to NMC of Ghana protocol guidelines for wound management.

Knowledge of the existing policy protocols in respect of wound management among nursing personnel is especially a challenge. It was found in this study (see Fig 2) that majority of nurses of all categories particularly had no knowledge of the existing policy protocol in respect of the use of methylated spirit and povidone iodine for non-surgical surgical wound management; use of normal saline and povidone iodine for non-surgical wound management, and when a trolley is bathed before wound management. Alhassan et al [12] and Welsh et al [26] alluded to similar findings on lack of routine in-service training and refresher courses for nurses as predominant reasons for this limited knowledge on current trends within the nursing profession.

Beyond the knowledge of existing policy protocols on standard nursing care, another challenge is the application of knowledge by those purporting to be conversant with the standard protocols of nursing care. In line with findings in some of the reviewed literature, it was found that even though more than two thirds of the staff demonstrated knowledge of standard policy protocols, similar to previous studies [7, 12, 25, 27], this knowledge did not translate into practice in terms of executing their duties. Alhassan et al [12] made similar observations when they discovered that even though nurses knew of the NMC policy guidelines for nasogastric tube feeding, majority of them, regrettably, did not apply this knowledge in the execution of their duties when independently observed.

In this study, it was also observed that a huge gap existed between self-rated/subjective scores of staff and the objective assessment ratings by independent assessors which were relatively lower. This observation is consistent with similar studies among nurses in Ghana [12, 28, 29] and other countries [30, 31]. Perhaps, social desirability responses by staff accounted for this discrepancy where respondents are more inclined to self-rate higher to possibly impress the researchers. Also, “strictness error” on the part of the independent assessors could have probably explained the lower objective scores. Nonetheless, this potential bias was largely controlled since the assessments were independently done by the trained research assistants.

These findings thus are a testament for future researchers to refrain from one-sided assessment of adherence from the view point of clinical staff since they turn to have higher inclination of providing socially desirable responses just to please researchers. A combination of objective and subjective clinical staff assessments on adherence to policy protocols is therefore advocated given this compelling empirical evidence demonstrated in this study.

In terms of the factors associated with nursing staff likelihood of adhering to standard nursing protocols, it was discovered that increasing years of work experience did not necessarily correlate positively with adherence to policy protocols in wound management. Indeed, increasing years of work experience had a negative association with adherence likelihood. This observation is contrary to previous studies where years of work experience associated with better professional practices by nurses and other clinical personnel [12, 26, 32]. Perhaps, the differences in methodology, cadre of respondents and clinical settings explain these variances in findings.

Unfortunately, the researchers did not chance on a tested theory that supports this rather counter intuitive observation. Nonetheless, anecdotal reports suggest unregulated task-shifting potentially induces this tendency of younger and lower ranks of nurses demonstrating better adherence to the standard protocols than their senior colleagues. Also, because lower cadre of nursing staff constituted a greater proportion of the study participants in this study their dominant views might have skewed the results. Large scale research designs in future will help understand these nuances and prefer better explanations to these observations.

Professional category of the respondents positively correlated with the likelihood of adherence to policy protocols for non-surgical and surgical wound management. It was found that professional nurses were more likely to adhere to the policy protocols relative to their auxiliary colleagues such as enrolled nurses. Previous studies have made similar conclusions on the association between higher professional ranks and compliance with standard clinical guidelines for nursing care [33]. However, a study by Alhassan et al [12] found contrary results when lower cadre of nurses were found to follow policy protocols for nasogastric tube feedings than higher professional ranks with the rebuttable argument perhaps, unregulated task-shifting and delegation of duties to lower rank nurses promotes mastery of the nursing skills and dexterity on the job over time by these lower ranks given that the nursing profession is also an art beyond being a science [33–35]. Additionally, staff with higher ranks are more likely to demonstrate higher likelihood of adherence than those with lower ranks, perhaps because the former might have been exposed to higher educational training and had better opportunities for in-service trainings and workshops on current policy protocols on wound management.

In this current study, the authors did not explore reasons for these revelations and would recommend future researchers consider employing blended qualitative and quantitative approach, to unearth reasons for these findings. Until this additional step is taken, conclusions would remain speculative and scientific guesses.

### Limitations

First, it is cautioned that the limited sample size of one out of sixteen (16) administrative regions of Ghana does not allow generalization of these findings to the general population as a true reflection of the situation in other health facilities and regions in Ghana. Future studies should therefore consider involving all sixteen regions and expanding the sample size to address this limitation. Furthermore, the objective assessments were performed for 30 out of the expected 140 respondents. In view of this limitation, the regression analysis was mainly run on the subjective responses sub-sample which has the potential of bias model output. Investigations in future could adopt better follow-up mechanisms, including longer study duration, to objectively assess respondents alongside their subjective reports.

## Conclusion

Study participants generally knew of the existing policy protocols for management of non-surgical and surgical wounds but this knowledge did not translate into actual adherence in terms of their practices in the clinical setting. Additionally, a wide variance was observed between subjective/self-ratings of respondents and the objective assessment scores by independent assessors. This revelation suggests, potential social desirability responses by the staff perhaps to impress the researchers. Finally, professional rank of staff other than years of work experience was a significant predictor of staff adherence to wound management protocols. These findings set the pace for future scientific investigators to employ multidisciplinary approach to generate in-depth knowledge on individual and health system level factors associated with adherence to standard policy protocols on wound management in health facilities in Ghana.

### Implications for health policy and clinical practice

While acknowledging the above limitations associated with this study, the following preliminary policy recommendations are proposed with the expectation that more upscaled investigations will adduce more empirical evidence for policy adoption of these recommendations:

1. Targeted continuous professional developments (CPDs) should be intensified through tailored made in-service trainings to suit the different professional ranks with particular interest in lower cadre of nursing staff who turn to be missed out in these CPDs
2. Additionally, licensing of healthcare facilities by regulatory agencies should consider policy reforms to incorporate routine staff-specific adherence to standard clinical protocols as pre-condition for licensing and renewal of health facilities.
3. Finally, enhanced bottom-up supervision of clinical staff will help in early identification of patient safety gaps and enforce policy protocols at the level of frontline clinical staff such as nurses.

## Supporting information

S1 File (DOCX)
