# Supplementary material for: Implementation of the policy protocol for management of surgical and non-surgical wounds in selected public health facilities in Ghana: An analytic case study
Source: PLoS One. 2020 Jun 23;15(6):e0234874. doi: 10.1371/journal.pone.0234874 (PMC7310699; doi:10.1371/journal.pone.0234874)
Supplement: S1 File — (DOCX) [file pone.0234874.s001.docx]

**UNIVERSITY OF HEALTH AND ALLIED SCIENCES, GHANA**

**Project Topic:** Implementation of the policy protocol for management of surgical and non-surgical wounds in selected public health facilities in Ghana: an analytic case study

**Preamble and informed consent**

***This survey investigates the extent to which clinical nursing staff adhere to the policy protocol for management of non-surgical and surgical wounds in selected public health facilities in Ghana. Your responses will be treated with privacy and confidentiality by anonymizing them. The interview will take approximately 30 minutes to complete after which an independent objective assessment where you would be observed performing tasks in surgical and non-surgical wound management and scored by two assessors. You can ask any question during the structured interview or the objective assessment. You also have the right to refuse to answer questions, or terminate your participation at any time without any consequences. If you have any further questions or concerns about this study, you may contact the Principal Investigator on (Tel +233-24-12-26-049 or e-mail:*** [*arkabason@gmail.com*](mailto:arkabason@gmail.com)***).***

***Do you declare to have understood the purpose of this survey and objective assessments and agree to voluntarily participate in this study?***

***YES [ ] NO [ ]***

***IF Yes, please sign here: …………………….……………………………………………………………………………………………………………..….***

**START TIME**_____________________________

**SECTION A: SOCIO-DEMOGRAPHIC AND WORK HISTORY OF PARTICIPANTS**

***Instruction:*** *Please chose the most applicable response to you in the second column and indicated the corresponding code in the third column.*

| **SN** | **Question** | **Response** | **Code** |
| --- | --- | --- | --- |
| 001 | Gender | Male_____________1  Female___________2 | \|  \| \| --- \| |
| 002 | Age | Please specify in years___________ | |
| 003 | Religion | Christianity________1  Islam_____________2  Other_____________3 | \|  \| \| --- \| |
| 004 | Professional rank | DDNS____________1  PNO_____________2  SNO_____________3  NO______________4  SSN_____________5  SN_______________6  PEN______________7  SEN______________8  EN_______________9  Other______________ | \|  \| \| --- \| |
| 005 | Professional category | RGN_____________1  RM______________2  RMN_____________3  RCN_____________4  NAC_____________5  NAP_____________6  Other_____________ | \|  \| \| --- \| |
| 006 | Years of practice as a nurse/auxiliary | Please specify______________ | |
| 007 | Years of work experience in facility | Please specify______________ | |

**SECTION B: KNOWLEDGE OF THE POLICY PROTOCOL FOR WOUND MANAGEMENT.**

***Instruction:*** *Please chose the most applicable response to you in the second column and indicated the corresponding code in the third column.*

| **SN** | **Question** | **Response** | **Code** |
| --- | --- | --- | --- |
| 001 | Are you aware there are standard practices in the dressing of septic wounds? | Yes___________1  No____________2 | \|  \| \| --- \| |
| 002 | How often do you revise the standard protocol for wound dressing in a year? | Please specify_________ |  |
| 003 | When do you bath the trolley? | Before dressing______1  During dressing______2  After dressing________3 | \|  \| \| --- \| |
| 004 | Do you ensure patients’ comfort during wound dressing? | Yes___________1  No____________2 | \|  \| \| --- \| |
| 005 | If Yes to question 004, please indicate how | Please specify_________ |  |
| 006 | How many times do you wash your hands during wound dressing? | Once______________1  Two times__________2  Three times_________3  Four times__________4 | \|  \| \| --- \| |
| 007 | At what times do you wash your hands during wound dressing? | Before dressing______________1  After removing old dressing____2  Before wearing surgical gloves__3  After wound dressing_________4 | \|  \| \| --- \| |
| 008 | Do you inform client about state of wound after dressing? | Yes___________1  No____________2 | \|  \| \| --- \| |
| 009 | Which solution do you use in non-surgical wound dressing? | Methylated spirit____________1  Povidone iodine____________2  Normal saline______________3  Antiseptic solution__________4 | \|  \| \| --- \| |
| 010 | Which solution do you use in surgical wound dressing? | Methylated spirit____________1  Povidone iodine____________2  Normal saline______________3  Antiseptic solution__________4 | \|  \| \| --- \| |

**SECTION C: ADHERENCE TO POLICY PROTOCOL FOR SURGICAL WOUND MANAGEMENT**

***Instruction:*** *How often do you follow these procedures anytime you are performing wound dressing for surgical wound? On a scale of 0-4. The interpretation of the parameters are as follows: 0 = very rarely adherent; 1 = rarely adherent; 2 = averagely adherent; 3 = often adherence; 4 = all the time adherent. Please indicate the most applicable perceived and observed adherence level.*

| **Standard criteria** | **Likert Scale** | | | | |
| --- | --- | --- | --- | --- | --- |
| **Surgical wound management** | **0** | **1** | **2** | **3** | **4** |
| 1. Establishes rapport and explains procedure to patient |  |  |  |  |  |
| 1. Put on mask, prepares and takes trolley to bedside and provides privacy |  |  |  |  |  |
| 1. Ask assistant to put patient into desired position, protect bed cloth and expose area. |  |  |  |  |  |
| 1. Ask assistant to pour out lotions into gallipot |  |  |  |  |  |
| 1. Ask assistant to remove plaster or bandage |  |  |  |  |  |
| 1. Remove soiled dressing with dissecting forceps or disposable gloves and discard |  |  |  |  |  |
| 1. Wash and dry hands and wear sterile gloves or use sterile forceps |  |  |  |  |  |
| 1. Clean wound with swaps soaked in normal saline using sterile forceps or sterile gloves |  |  |  |  |  |
| 1. starting from the wound outward using one swap at a time. |  |  |  |  |  |
| 1. Cleans wound with series of swaps until clean |  |  |  |  |  |
| 1. Apply sufficient sterile dressing and secure into position |  |  |  |  |  |
| 1. Inform patient about state of wound |  |  |  |  |  |
| 1. Thank and make patient comfortable in bed |  |  |  |  |  |
| 1. Discard trolley, decontaminate used items and remove gloves |  |  |  |  |  |
| 1. Wash and dry hands and remove screen |  |  |  |  |  |
| 1. Document and report state of wound |  |  |  |  |  |

**SECTION D: ADHERENCE TO POLICY PROTOCOL FOR NON-SURGICAL WOUND MANAGEMENT**

***Instruction:*** *How often do you follow these procedures anytime you are performing wound dressing for surgical wound? On a scale of 0-4. The interpretation of the parameters are as follows: 0 = very rarely adherent; 1 = rarely adherent; 2 = averagely adherent; 3 = often adherence; 4 = all the time adherent. Please indicate the most applicable perceived and observed adherence level.*

| **Standard criteria** | **Likert Scale** | | | | |
| --- | --- | --- | --- | --- | --- |
| **Non-surgical wound management** | **0** | **1** | **2** | **3** | **4** |
| 1. Explain procedure to patient and ensures privacy |  |  |  |  |  |
| 1. Prepare and take trolley to the patient’s bedside |  |  |  |  |  |
| 1. Position patient comfortably and protect bedclothes |  |  |  |  |  |
| 1. Expose area of wound and remove plaster or bandage |  |  |  |  |  |
| 1. Wash and dry hands, assemble instruments and pour lotions into gallipots |  |  |  |  |  |
| 1. Remove soiled dressing with dissecting forceps or gloved hand, discard, wash and dry hands |  |  |  |  |  |
| 1. Dab or clean wound with sterile forceps or gloves using prescribed lotion |  |  |  |  |  |
| 1. Irrigate cleaned wound with syringe and saline from within outward and clean the surrounding skin |  |  |  |  |  |
| 1. Clean wound with series of swaps until its clean |  |  |  |  |  |
| 1. Apply sterile dressing using prescribed dressing lotion and secure into position or leave exposed where necessary |  |  |  |  |  |
| 1. Make patient comfortable in bed, explain relevant findings to patient and thank him |  |  |  |  |  |
| 1. Discard trolley and decontaminate used instruments and wash hands |  |  |  |  |  |
| 1. Remove gloves and screen, wash and dry hands |  |  |  |  |  |
| 1. Document and report state of the wound |  |  |  |  |  |

**SECTION E: PERCEIVED CHALLENGES AND CONTRAINTS IN ADHERING TO POLICY ON WOUND MANAGEMENT**

***Instruction:*** *Please indicate in the spaces the most encountered challenges confronting you in adhering to policy protocol for surgical and non-surgical wound management*

………………………………………………………………………………………………………………………………………………………………………………………………………………………………………………………………………………………………………………………………………………

………………………………………………………………………………………………………………………………………………………………………………………………………………………………

………………………………………………………………………………………………………………………………………………………………………………………………………………………………………………………………………………………………………………………………………………

………………………………………………………………………………………………………………………………………………………………………………………………………………………………

………………………………………………………………………………………………………………………………………………………………………………………………………………………………………………………………………………………………………………………………………………

………………………………………………………………………………………………………………………………………………………………………………………………………………………………………………………………………………………………………………………………………………………………………………………………………………………………………………………………………………………………………………………………………………………………………………

………………………………………………………………………………………………………………………………………………………………………………………………………………………………………………………………………………………………………………………………………………………………………………………………………………………………………………………………………………………………………………………………………………………………………………

………………………………………………………………………………………………………………………………………………………………………………………………………………………………………………………………………………………………………………………………………………………………………………………………………………………………………………………………………………………………………………………………………………………………………………

………………………………………………………………………………………………………………………………………………………………………………………………………………………………………………………………………………………………………………………………………………………………………………………………………………………………………………………………………………………………………………………………………………………………………………

………………………………………………………………………………………………………………………………………………………………………………………………………………………………………………………………………………………………………………………………………………………………………………………………………………………………………………………………………………………………………………………………………………………………………………

**THANK YOU**

**END TIME**________________________________________________
